# Scope and cost-effectiveness of fermented corn straw roughage-based buffalo fattening approach

**DOI:** 10.5455/javar.2021.h502

**Published:** 2021-05-19

**Authors:** Biplob Kumer Roy, Nazmul Huda, Nasrin Sultana

**Affiliations:** Animal Production Research Division, Bangladesh Livestock Research Institute, Savar, Dhaka, Bangladesh

**Keywords:** ADG, buffalo, fattening, FCM, FCR, profitability

## Abstract

**Objective:**

This experiment was undertaken to assess the scope and cost-effectiveness of the fermented corn mixture (FCM)-based buffalo fattening approach compared to urea molasses straw (UMS) and silage-based approach.

**Materials and Methods:**

A completely comparative randomized design experiment was conducted for 90 days with three treatments and five buffalo bulls in each. UMS, silage, and FCM roughage-based fattening diets were attributed as T_1_, T_2_, and T_3_, respectively. Two types of protein supplements, i.e., Type 1 (Bangladesh Livestock Research Institute-developed) for T_1_ and T_2 _and Type 2 (prescribed by farmers) for T_3 _treatments, were used. All the parameters were analyzed through Statistical Package for the Social Sciences, 20 software.

**Results:**

Dry matter intake (kg, %live weight) was significantly higher in T_1_ (2.65), followed by T_2_ (2.34) and T_3_ (2.00), respectively. The crude protein intake, digestible crude protein intake, and digestible dry matter intake (kg/d) significantly (*p* < 0.05) differed between T_1_ and T_3_, but not T_2_. The digestibility of acid detergent fiber (65.97) was significantly higher for T_3_ than T_1_ and T_2_ (54.44 and 58.73, respectively). Neutral detergent fiber digestibility of T_3_ (70.35) also differed (*p* < 0.05) with T_1_ (60.97) but not T_2_ (64.78). No difference was observed in the case of growth, but feed conversion ration was found to be significantly (*p *< 0.05) better in T_2_ (7.10) than T_1_ (8.35), where T_3_ (7.24) was neutral. The significantly (*p* < 0.001) highest expense [216.37 Bangladesh taka (BDT)/kg gain] was required for T_1_, followed by T_2_ and T_3_ (174.47 and 126.33 BDT/kg gain, respectively). Net profit from T_3_ and T_2_ (15,877 and 15,175 BDT, respectively) gained significantly (*p* < 0.05) higher than T_1_ (11,265 BDT).

**Conclusion:**

The FCM-based diet was suitable and cost-effective as a buffalo fattening approach.

## Introduction

Buffalo, a promising domestic animal, is a dedicated resource of potential meat production in South Asia. Considering the region of Asia, 79.74% of buffaloes are available in South Asian countries, and the remaining 20.26% in other countries of Asia [1]. It is well known that buffalo farming is labor-intensive and cost-effective. Buffaloes have milk and meat-producing entities by origin, and if fed well at the growing stage, they are capable of producing supreme meat with efficient costing. They are remarkable for their ability to feed conversion with very low input. Even they utilize the straw or any kind of coarse fibrous feed suitably in their body by converting it in to quality meat [2].

In comparison, the observed daily weight gain and feed conversion ratio of buffalo are similar to cattle [3]. They are also appreciated for their disease resistance, flexible feeding, and well response in varietal farming conditions [4]. They performed excellently in the live weight gain (LWG) and the feed conversion ratio [5]. The main features of buffalo meat are that the meat is red, contains a high amount of protein but low fat and cholesterol with little marbling, the presence of expected meat texture, shows a capacity of emulsifying and water holding as well [6]. 

The type of buffalo in Bangladesh is indigenous, and the riverine type is prominent in number. In the eastern part of the country, swamp-type buffaloes are found. Surrounding the Indian border, the crossbred buffalo of Murrah, Nili-Ravi, Surti, and Jaffrabadi are also available [7]. According to the data of DLS [8], 1.464 million buffaloes are available in Bangladesh. Bangladesh’s livestock production system is mainly based on small farming, with 67.1% of total livestock [9], where they largely depend on insufficient livestock feed resources.

In most cases, livestock farmers mainly use poor quality straw or roughage as a basal diet because of its widespread availability and low cost [10]. Through simple dietary manipulations, the existing feed stuff can improve a lot, and numerous opportunities can be created in enhancing an animal’s performance. Just adding urea with straw at a certain amount, the crude protein content of straw increased by 2.8%–6.5% intently [11]. 

Urea-treated straw or urea molasses-treated straw is widely used in the country for fattening or dairying. In the field, it is proven as farmer-friendly and the most appropriate method for farmers from a fattening point of view [12]. Moreover, by using urea molasses-treated straw in many countries, farmers obtain better performance from ruminants than conventional feeding [13]. Because ruminant animals have absolute potential to modify the dietary nitrogen (N) into a human’s consumable good quality protein like meat [14], besides this approach, making fodder palatable by ensiling and using it for fattening enterprises is also a common and popular technique. With moderate protein supplementation, good quality silage fodder could perform best in fattening enterprises [15,16]. 

Another modern technique is the total mixed ration (TMR), which is widely served for fattening or other livestock farming purposes. It is a complete mixture of roughage, grains, protein supplement, feed additives, vitamins, and minerals; actually, it consists of all types of feed ingredients. It is proven that by using the TMR (the mixture of concentrate and forest grass), supplementation, dry matter (DM), protein, and energy intake of crossbred calves improved. TMR is used in case of intensive feeding. In 1999, quality buffalo meat production from male buffalo was started in commercial feedlots by rearing them with intensive feeding [4]. At that time, yearling buffalo bulls were purchased from farmers and were fed a high energy and protein-rich diet for fattening for 4 months. During that period, the yearling buffalo bulls grew from 0.9 to 1.0 kg/day at least and gave an impressive dressing percentage (DP) [17]. 

However, the modern approach of the present era is different from the previous fattening technique. Nowadays, in fattening feeding, the manipulation of roughage to concentrate ratio (R:C) is required and notably affects the weight gain and feed conversion efficiency of animals. Over time, this approach has changed a lot and has focused on increasing more and more grain feeding. This trend is primarily followed in feedlot diets. A good weight gain is observed when feeding ration in a roughage:concentrate ratio (R:C) of = 25:75 with little risk. Although this ratio changes with different proportions by increasing concentrate (from R:C = 50:50 to R:C = 10:90), it will also be applicable [18]. The fact was that by increasing forage concentration in the diet the only intake of DM increased but did not reflect on the average daily gain (ADG) of animals, resulting in a high feed conversion ration (FCR) with low ADG [19]. 

However, the main constraint of no fattening or poor performance of buffalo bulls in most parts of Asia is the unavailability of adequate and available quality feed stuff. The rearing of buffaloes in this region depends on cereal straw (highly lignified) or grazing in low-quality pasture land. They can harvest only a small amount of fermentable protein, which is insufficient for their growth or even maintenance. Over the years, many buffalo fattening approaches have been invented and adopted to improve fattening diets and improve the degree of utilization. As a result, many types of buffalo fattening approaches exist in the field. Some of them are urea molasses straw (UMS)-based, some silage or TMR, or any other roughage-based. Nowadays, in Bangladesh, fermented corn mixture (FCM) feed is used for fattening without measuring the actual efficacy. The time has come to identify the most suitable fattening approach, which can be run with a low cost and readily available resources. Thus, the present investigation was conducted to evaluate the scope and cost-effectiveness of an FCM roughage-based diet for fattening the buffalo compared with the UMS and silage-based fattening approaches. 

## Materials and Methods

The methodology of this experiment was approved by the Animal Experimentation Ethics Committee of Bangladesh Livestock Research Institute (BLRI) with an approval number BLRI/0011 (dated: 10 November 2020).

A completely randomized design (CRD) experiment was carried out at the BLRI for 90 days, including 7 days of conventional digestion trial, where 15 local growing river-type water buffalo bulls (2–3 years of age) were enshrined in three treatments having five animals each. All the animals were housed individually in separate pens and provided feed in two equal meals at 9:00 and 16:00 h. Before the feeding trial, animals were dewormed properly with anthelmintics (Levamesol BP 600 mg per bolus) and were maintained for 10 days for adjustment and for calculating the DM requirements. Regarding roughage, UMS, silage, and FCM-based fattening diets were specified for T_1_, T_2_, and T_3_, respectively, and a comparative study was accomplished for exploring the best practice.

Two types of protein supplements were used for three treatments. BLRI-developed protein supplement formula was selected as “Type 1” and used for UMS and silage-based conventional fattening. A new formula of making protein supplement (Type 2) prescribed by farmers was used for FCM-based fattening practice. In the case of T_1_ and T_2_ treatments, based on the DM requirement of each animal, approximate 40% of the DM (1% of their body weight) was supplied as a protein supplement (Type 1), and the rest of the 60% (1.5% of their body weight) of the DM was provided as roughage. An additional amount of feed was provided as roughage if required. In the case of T_3_, based on the DM requirement of each animal, approximate 50% of the DM (1.25% of their body weight) was supplied as FCM, and 10% of the DM (0.25% of their body weight) was provided as a protein supplement (Type 2). The rest of the 40% (1% of their body weight) required DM was provided as roughage. An additional amount of feed was provided as roughage if needed. UMS was used for T_1_ treatment and Napier silage was used for T_2_ and T_3_ treatments. UMS was made regularly according to the method of Huque and Chowdhury [20]. 

Napier (Packchong) fodder was ensiled in a trench silo for 21 days with ensuring anaerobic condition, and produced silage was used. Like silage, FCM is also made from corn powder, water, rice straw, molasses, and urea at 40%, 40%, 15%, 4%, and 1% proportion of combination, respectively. Type 1 protein supplement composed of broken wheat, wheat bran, khesari bran, soybean meal, *dicalcium phosphate* (DCP), limestone, salt, and vitamin–mineral premix at 20%, 40%, 20%, 15%, 3%, 0.9%, 1%, and 0.1% level of inclusion, respectively, and Type 2 protein supplement composed of soy meal, corn powder, salt, DCP, and limestone at 50%, 35%, 5%, 5%, and 5% level of inclusion, respectively (Table 1). 

Animals were weighed at an interval of 10 days during the whole trial period. The DM, organic matter (OM), and crude protein (CP) content were determined following the Association of Official Analytical Chemist [21], and acid detergent fiber (ADF) content and neutral detergent fiber (NDF) content were determined according to the procedure described by Van Soest et al. [22]. The apparent digestibility coefficients for DM, OM, CP, ADF, and NDF were calculated from a constituent’s dietary intake and the amount recovered in feces. The gross energy [GE; Megajoule (MJ)/kg DM] of feed samples was measured through an adiabatic bomb calorimeter (Model no. IKA*C5000). The DP of buffalo bulls was estimated as 53%, according to the report of Naveena and Kiran [23]. Growth performance, feed intake, digestibility of nutrients, FCR, and cost-net profit calculation were analyzed statistically using the analysis of variance of a CRD using the compared means with Statistical Package for the Social Sciences, 20 software package. 

## Results

The fresh biomass of UMS, Napier (Packchong) silage, FCM, and the concentrate mixture of Type 1 and 2 contained 57.80%, 18.46%, 61.79%, 87.73%, and 89.18% DM, respectively (Table 2). Between two fresh roughages, crude protein (9.47% and 7.98% DM, respectively) and GE (20.63 and 16.00 MJ/kg DM, respectively) were higher in UMS than Napier silage. On the contrary, CP percentage and GE of FCM were 15.91% DM and 24.13 MJ/kg DM, which was almost the standard of concentrate. The notable thing was that costing increased when CP and GE got higher [cost = 11.60, 6.50, and 14.71 Bangladesh taka (BDT)/kg DM, respectively]. Two types of concentrate showed the results differently, where more CP and GE were found along with the low cost. In Type 1 and 2 concentrate mixtures, 18.21 and 22.92 (%DM) CP content and 16.48 and 24.26 volume of GE (MJ/kg DM) was found along with the expense of 36.00 and 33.42 BDT/kg DM, respectively. The ADF content of UMS, Napier silage, FCM, and two types of concentrate were 42.82%, 59.06%, 15.18%, 14.53% and 10.65%, respectively, and NDF percentage was 67.24, 86.92, 49.54, 31.34 and 30.18, respectively. Simultaneously, GE (MJ/kg DM) of UMS, Napier silage, FCM, and two types of concentrate were 20.63, 16.00, 24.13, 16.48 and 24.26, respectively. Another notable thing was that the lower amount of ADF-rich diets showed a higher GE production volume (Table 2). 

**Table 1.** The proportion of ingredients of protein supplements and FCM.

Protein supplement; Type 1 (BLRI developed)		Protein supplement; Type 2(Farmer’s practice)		FCM	
Ingredients	Inclusion level (%)	Ingredients	Inclusion level (%)	Ingredients	Inclusion level (%)
Broken wheat	20	Soy meal	50	Corn powder	40
Wheat bran	40	Corn powder	35	Water	40
Khesari bran	20				
Soybean meal	15	Salt	5	Rice straw	15
DCP	03				
Limestone	0.9	DCP	5	Molasses	4
Common salt	01				
Premix	0.1	Limestone	5	Urea	1

Based on the live weight percentage of animals, UMS-based diet treatment ingested a much more significant percent (*p* < 0.001) of DM [2.65 kg of %live weight (LW)], followed by silage and FCM-based diets (2.34 and 2.00 kg of %LW, respectively). When total DM intake was diminished and differed significantly among the treatments, most of the other nutrient intake measurements showed the same trend keeping the highest value with UMS-based diet. The crude protein intake (CPI) (kg/day) differed significantly (*p *< 0.05) among the treatments, and T_1_ and T_3_ differed with each other (1.03 and 0.75 kg/day, respectively), but T_2_ (0.89 kg/day) did not differ with any of them. Then, the value of digestible CPI also differed in the same manner. Although the dry matter intake (DMI) did not show any significant difference among the treatments, digestible dry matter intake (DDMI) was found to be higher (5.18 kg/day) with T_1_ and significantly differed (*p* < 0.05) with T_3_ (3.98 kg/day), whereas T_2_ (4.57 kg/day) had no difference with others. In nutrient digestibility, no significant difference was observed with DM, OM, and CP digestibility. 

Because of the lower content of ADF in diets, the FCM-based diet group showed significantly higher (*p* < 0.001) digestibility (65.97) than the silage and UMS-based diet group (58.73 and 54.44, respectively). The NDF digestibility showed almost similar results with little difference, whereas the FCM-based diet group (70.35) significantly differed (*p* < 0.05) with UMS-based diet group (60.97), but silage-based diet group (64.78) did not differ significantly from any others. Before the experiment, the animals were distributed in groups as they had no initial live weight difference among the treatments. After 90 days of trial, again, the measurement of the final live weight and even ADG of animals did not perform differently among the treatments. Compared to other treatments, bulls of silage-based diet showed significant (*p* < 0.05) better feed conversion efficiency. A significant difference was observed between silage (7.10) and UMS-based diet (8.35). There was no significant difference observed between FCM (7.24) and silage or FCM and UMS-based diet (Table 3). 

**Table 2.** The chemical composition of different diets and their costing.

Items	DM, % of fresh biomass	Chemical composition (%DM)				GE (MJ/kg DM)	Cost (BDT/kg DM)
		OM	CP	ADF	NDF		
UMS	57.80	85.50	9.47	42.82	67.24	20.63	11.60
Napier (packchong) silage	18.46	93.00	7.98	59.06	86.92	16.00	6.50
Fermented corn mix.	61.79	91.51	15.91	15.18	49.54	24.13	14.71
Conc. mix. (Type 1)	87.73	92.25	18.21	14.53	31.34	16.48	36.00
Conc. mix. (Type 2)	89.18	83.34	22.92	10.65	30.18	24.26	33.42

DM = Dry matter; OM = Organic matter; CP = Crude protein; ADF = Acid detergent fiber; NDF = Neutral detergent fiber; GE = Gross energy.

The cost of different diet types for per kg *LWG* of buffalo bulls was identified separately, and then all the results were accumulated, and the total cost involvement was determined. For making UMS and FCM, an extra cost was required, which reflects in results. Significantly (*p* < 0.001) lower costing was required for silage-based roughage (26.08 BDT/kg gain) than UMS (59.80 BDT/kg gain) and FCM-based roughage (59.80 BDT/kg gain). Since the FCM-based diet group obtained a little amount of concentrate than others, then very logically, the costing of protein supplement of FCM-based treatment (27.20 BDT/kg gain) differed significantly (*p* < 0.001) with UMS and silage-based treatments (114.8 and 111.2 BDT/kg gain). The roughage, concentrate, and refusal costs were combined and expressed as total feed cost, where a significant difference was observed (*p* < 0.001) among the treatments. The total feed costing was higher with the UMS-based diet group (181.37 BDT/kg gain), followed by the silage and FCM-based diet groups (141.46 and 91.35 BDT/ kg gain, respectively). After inclusion of management cost, the total costs per kg *LWG* showed similar results, whereas the costing of T_1_ was significantly (*p* < 0.001) higher (216.37 BDT/ kg gain), followed by T_2_ and T_3_ (174.47 and 126.33 BDT/kg gain) (Table 4).

**Table 3.** Nutrient intake, digestibility, growth, and FCR estimation of buffalo bulls.

Parameters	Treatments			SED	Sig.
	T_1_	T_2_	T_3_		
DMI (kg/day)	8.07	7.12	6.15	0.50	NS
DMI (kg; % LW)	2.65^a^	2.34^b^	2.00^c^	0.03	***
OMI (kg/day)	7.11	6.60	5.62	0.42	NS
CPI (kg/day)	1.03^a^	0.89^ac^	0.75^bc^	0.06	*
ADFI (kg/day)	2.49	2.75	2.43	0.19	NS
NDFI (kg/day)	4.25	4.45	4.25	0.32	NS
DDMI (kg/day)	5.18^a^	4.57^ac^	3.98^bc^	0.25	*
DOMI (kg/day)	4.82	4.39	3.75	0.30	NS
DCPI (kg/day)	0.69^a^	0.60^ac^	0.51^bc^	0.04	*
DM digestibility	64.07	64.19	65.08	0.61	NS
OM digestibility	67.72	66.49	66.96	0.59	NS
CP digestibility.	66.62	66.87	67.79	0.64	NS
ADF digestibility	54.44^b^	58.73^b^	65.97^a^	1.29	***
NDF digestibility	60.97^b^	64.78^bc^	70.35^ac^	1.54	*
Initial LW(kg)	259.20	256.60	257.80	20.82	NS
Final LW (kg)	346.77	346.80	333.24	24.43	NS
ADG (kg)	0.97	1.00	0.84	0.05	NS
FCR	8.35^a^	7.10^bc^	7.24^ac^	0.32	*

Means within columns bearing different superscripts differ significantly.

NS = Non significant; DMI = Dry matter intake; OMI = Organic matter intake; CPI = Crude protein Intake; ADFI = Acid detergent fiber intake; NDFI = Neutral detergent fiber intake; DDMI = Digestible dry matter intake; DOMI = Digestible organic matter intake; DCPI = Digestible crude protein Intake; ADG = Average daily gain; FCR = Feed conversion ratio.

* *p* < 0.05;

*** *p* < 0.001;

Total LWG of the whole experimental period was 87.57, 90.20, and 75.44 kg (T_1_, T_2_, and T_3_, respectively). To gain these amounts of live weight, the feed cost for UMS-based diet group (15,754 BDT) was significantly (*p* < 0.001) higher than the silage and FCM-based diet group (12,750 and 6,961 BDT, respectively). Again, after the inclusion of management cost, total cost showed a similar trend. Carcass weight was determined from total LWG, and estimated the DP of T_3_ showed lower carcass weight (40 kg) than T_1_ and T_2_ (46.4 and 47.8, respectively). The carcass price was measured with 500 BDT/kg, and the cost of the non-carcass portion was measured with 150 BDT/kg (at 30% of the carcass’ price). Everywhere, T_3_ showed the lower price of the carcass and non-carcass portions than other treatments. Then, the total selling price was fixed as 30,169, 31,075, and 25,988 for UMS, silage, and FCM-based diet, respectively. At last, when net profit was observed, a more significant profit (*p* < 0.05) was obtained from FCM and silage-based diet groups (15,877 and 15,175 BDT, respectively) than UMS-based diet group (11,265 BDT) (Table 5). 

## Discussion

The UMS was prepared following the guidelines of Huque and Chowdhury [20], and the result of this experiment found a resemblance with their study, wherein they found 10.00% CP and 40.60% ADF in UMS, and 9.47% CP and 42.82% ADF were found in this study. The FCM-based diet technology has a lot of similarity to the TMR-based diet technology, which is practiced sporadically mainly in Bangladesh. So, it was required to find out the efficacy of this feeding technique. The FCM-based feeding technique was found somewhat similar to Hoque et al. [24]. The crude protein content of UMS, Napier silage, and concentrate; Type 1 and 2 (9.47%, 7.59%, 18.21% and 22.92% DM basis, respectively) were genuinely compatible with the results of Hoque et al. [24], where they got 10.06%, 7.59%, and 22.65% DM (%DM basis) in urea-treated straw, green fodder, and concentrate, respectively. 

**Table 4.** Cost (BDT) involvement in different diets for per kg LWG of buffalo bulls.

Cost (BDT/per kg gain)	Experimental diets				
	T_1_	T_2_	T_3_	SED	Sig.
Roughage	59.80^a^ ± 3.07	26.08^b^ ± 0.88	59.80^a^ ± 3.81	2.35	***
Protein supplement	114.8^a^ ± 5.48	111.20^a^ ± 7.28	27.20^b^ ± 1.65	4.37	***
Refusal	6.77^a^ ± 0.80	4.20^ac^ ± 0.71	4.18^bc^ ± 0.74	0.61	*
Total feed	181.37^a^ ± 8.54	141.46^b^ ± 8.59	91.35^c^ ± 5.12	6.20	***
Management	35.00 ± 0.00	35.00 ± 0.00	35.00 ± 0.00	0.00	NS
All Total	216.37^a^ ± 8.54	176.46^b^ ± 8.59	126.33^c^ ± 5.12	6.20	***

Means within columns bearing different superscripts differ significantly.

* = *p* < 0.05,

*** = *p* < 0.001, NS = Non-significant.

**Table 5.** The total expenditure of different experimental diets and benefit in return.

Parameters	T_1_	T_2_	T_3_	SED	Sig.
LWG (kg)	87.57 ± 6.44	90.20 ± 5.34	75.44 ± 6.42	4.97	NS
Feed cost (BDT)	15,754^a^ ± 964	12,750^b^ ± 1,005	6,961^c^ ± 842	767	***
Management cost	3,050 ± 0.00	3,050 ± 0.00	3,050 ± 0.00	0.00	NS
Total cost	18,904^a^ ± 964	15,900^b^ ± 1,005	10,111^c^ ± 842	767	***
Carcass wt (kg; DP% 53)	46.40 ± 3.47	47.80 ± 2.73	40.00 ± 3.39	2.62	NS
Price of the carcass (BDT 500)	23,207 ± 1,709	23,904 ± 1,416	19,991 ± 1,700	1,318	NS
Price of the edible/ non-edible portion (at 30% of the carcass price; BDT 150)	6,962 ± 513	7,171 ± 425	5,997 ± 510	395	NS
Total Selling price (BDT; at 500)	30,169 ± 2,221	31,075 ± 1,840	25,988 ± 2,210	1,713	NS
Net profit (BDT; at 500)	11,265^b^ ± 1,585	15,175^a^ ± 1,400	15,877^a^ ± 1,472	1,215	*

Means within columns bearing different superscripts differ significantly.

* *p* < 0.05;

*** *p* < 0.001.

NS = Non-significant; LWG = Live weight gain.

Burque et al. [25] obtained 10.41% crude protein in UMS, which is almost similar to this experiment’s result. The cattle fattening program conducted by Saha et al. [26] observed that the average DM and CP intake of native bulls was 4.75 and 0.53 kg/day. Their digestibility was 63.89% and 71.62%, respectively, under the UMS-based diet. In this experiment, by applying the same diet, DM and CP intake were 8.07 and 1.03 kg/day, double the previous experiment with 64.07% and 66.62% digestibility. The variations of feed intake might be happened because of species differences. In this regard, Terramoccia et al. [27] reported that the CP and protein-free DM degradation rate is better in buffalo than cattle. The difference in CPI (kg/day) was observed among the treatments because of the amount of protein supplements. Bulls of FCM-based treatment only received 0.25% of protein supplement of their body weight, whereas others received 1%. At the same time, UMS contained more protein than Napier silage. 

In a comparative experiment on crossbred cattle and buffalo, Lapitan et al. [28] found a higher DM and CP intake with buffalo fed Napier silage (6.73 and 0.66 kg/day, respectively), which is almost similar to the present study (7.12 and 0.89 kg/day, respectively). On the measure of percent live weight, DMI was higher in UMS-based diet, followed by silage-based diet and FCM-based diet. This may happen for the palatability of UMS, as described by Sarwar et al. [29]. FCM, a maiden diet, may also influence the DMI kg/percent LW. However, it is observed that, by feeding ammonia-treated wheat straw, feed intake, its digestibility and milk production of buffaloes are increased [30]. There was a potent myth in South Asian buffalo farmers; they believe buffalo productivity may hamper silage feeding [31]. But, in this experiment, no deleterious effect was observed by silage feeding. This statement was compatible with the study of Touqir et al. [32], where they found that the jamboo or mott grass silage could be used safely as an alternative to conventional grass without affecting nutrient digestibility. This experiment observed that when any feed contained a low amount of ADF, it produced a high GE volume. This statement has drawn a coherence with Rasby et al. [33], where they mentioned that forage with low ADF concentrations is higher in energy. Although there were three treatments, two types of grasses were used in this study, and it revealed that, with the increment of digestibility, the NDF intake increased, which is supported by the statement of Rasby et al. [33]. 

In the case of ADG of buffalo bulls, Naveena and Kiran [23] stated that, within the conventional farming system, buffalo bulls increased 0.39–0.54 kg/day in the Indian regional context. Lapitan et al. [28] stated that, in the Philippines, the crossbred water buffalo bulls gained up to 0.49 kg per day when they were fattened with high roughage diet (only 15% concentrate). In this experiment, applying the fattening approach, it is observed that it is achievable with different diet types by 0.84–1.00 kg/day of ADG. The result resembles Burque et al. [25] findings, where they achieved a maximum of 0.91 kg *LWG* per day from fattened buffalo calve with straw-based ration in Pakistan. Cattle and the Brahman crossbred bulls also gained a similar bodyweight (0.95–0.98 kg/day) with concentrate-rich diets [34]. The best feed conversion ratio was found with Napier silage-based diet (7.10), but Lapitan et al. [28] observed much more FCR (14.8) with the same types of diet. 

However, the result of this experiment resembled the study of Rashid et al. [34], where Brahman crossbred bulls showed 6.76–7.04 FCR with different diets. In this experiment, the three treatment costs varied because of the difference in feed ingredients – making and processing UMS and FCM tools more expensive than silage. Again, T_3_ received a low amount of concentrate because of little protein supplement allocation, which tools less costly than others. As a result, all total costs differed significantly from each other. The DP (53%) was estimated following Naveena and Kiran’s [23] suggested standard, which was based on this sub-continent water buffalo. Another study stated that the DP of buffalo ranges from 55.4 to 59.0 when reared with a moderate diet. At the same time, veal yields were observed to be 61%–64% [35]. Finally, in return, more expense of T_1_ also reflects in the net profit margin, whereas it was observed that the silage and FCM-based diet is more profitable (*p* < 0.05) than the UMS-based diet.

## Conclusion

In the end, all the comparisons reflected that the FCM roughage-based diet could be used for buffalo fattening enterprises. Because of its performance in buffalo fattening, there has a great scope to use this technique. The cost-effectiveness of this diet was also preferable. So, the FCM-based diet could be used as a suitable buffalo fattening approach. 

## List of abbreviations

FCM = Fermented corn straw, UMS = Urea molasses straw, LW = Live weight, DM = Dry matter, OM = Organic matter, CP = Crude protein, ADF = Acid detergent fiber, NDF = Neutral detergent fiber, GE = Gross energy, DMI = Dry matter intake, CPI = Crude protein intake, DDMI = Digestible dry matter intake, DCPI = Digestible crude protein intake, CPI = Crude protein intake, ADG = Average daily gain, FCR = Feed conversion ratio, BDT = Bangladesh taka, BLRI = Bangladesh livestock research institute, TMR = Total mixed ration, DCP = Dicalcium phosphate, LWG = Live weight gain, kg = Kilogram, MJ = Megajoule, R = Roughage, C = Concentrate, DP = Dressing percentage
